# Comparison of glaucoma diagnostic ability of ganglion cell-inner plexiform layer according to the range around the fovea

**DOI:** 10.1186/s12886-019-1283-y

**Published:** 2019-12-30

**Authors:** Jae Ho Jung, Je Hyun Seo, Min Seung Kang, Jonghoon Shin

**Affiliations:** 1 Department of Ophthalmology, College of Medicine, Seoul National University Hospital, Seoul, South Korea; 2 Department of Ophthalmology, Veterans Medical Research Institute, Veterans Health Service Medical Center, Seoul, South Korea; 3 Department of Ophthalmology, College of Medicine, Pusan National University Yangsan Hospital, Yangsan, South Korea; 4 Department of Ophthalmology, Research Institute for Convergence of Biomedical Science and Technology, Pusan National University Yangsan Hospital, Yangsan, South Korea; 5 Department of Ophthalmology, School of Medicine, Pusan National University Yangsan Hospital, Pusan National University, 20-Geumo-ro, Mulgeum-eup, Yangsan, 50612 South Korea

**Keywords:** Glaucoma, Ganglion cell-inner plexiform layer, Wide-angle swept-source OCT

## Abstract

**Background:**

To compare the glaucoma diagnostic ability of the ganglion cell-inner plexiform layer (GCIPL) thickness depending on the range around the fovea using wide-angle, swept-source optical coherence tomography (SS-OCT).

**Methods:**

We compared the glaucoma diagnostic utility of GCIPL parameters across multiple regions while centered on the fovea. In a wide-angle scan, the GCIPL for each 1-mm^2^ grid square of a 12 × 9 mm^2^ scan resulted in 108 data points. With respect to the range of the GCIPL measurements around the macula, the wide-angle scan images were classified into three zones. Zone 1 was defined as a narrow area; zone 2 was defined as a mid-sized area; and zone 3 was defined as a wide area. We recorded the quadrant GCIPL thickness, average, and minimum quadrant GCIPL within each zone. The areas under the receiver operating characteristic (AUROCs) curves were calculated to evaluate the glaucoma diagnostic utility.

**Results:**

Sixty-one eyes with glaucoma and 59 normal eyes were assessed. The minimum and average GCIPL measurements in zones 1–3 in eyes with glaucoma were significantly lower than those in normal eyes (*P* <  0.001). The AUROCs for the minimum and inferotemporal GCIPL in zone 1 and the inferotemporal GCIPL thickness in zone 2 were greater than 0.9 (0.945, 0.931, and 0.918, respectively).

**Conclusions:**

Wide-angle scanning using SS-OCT will contribute to improvements in the detection of glaucomatous damage. The minimum and inferotemporal GCIPL in zone 1 may be more useful for detecting glaucoma than those in the conventional area.

## Background

Glaucoma is characterized by the degeneration of ganglion cells, alterations to the optic nerve head morphology, and associated visual field (VF) loss [1, 2]. Previous studies have indicated that considerable damage to retinal ganglion cells can precede VF loss in patients with suspected glaucoma and that ganglion cell-inner plexiform layer (GCIPL) parameters are a useful tool in the diagnosis of glaucoma [3–6].

Recently, GCIPL assessment with commercially-available spectral domain optical cohrerence tomography (SD-OCT) tools has become a standard clinical approach; however, this technique has several limitations with respect to the precise GCIPL parameter measurements that are needed to diagnose glaucoma. This protocol relies on the use of a fixed, 6-mm-diameter circular device that was developed not to measure GCIPL thickness but rather to diagnose and monitor significant macular edema in cases of diabetic retinopathy. Therefore, conventional GCIPL measurement may have limited diagnostic sensitivity and specificity when the GCIPL loss is less than or greater than 6 mm in diameter.

Swept-source OCT (SS-OCT), a new generation of OCT, was recently developed. This technique allows clinicians to obtain a high-quality, wide-angle image that includes coverage of the optic disc and macula and has a rapid scan speed. The SS-OCT technique enables GCIPL parameter calculations for each 1-mm^2^ grid square across a 12 × 9 mm^2^ scan, resulting in 108 data points that can be displayed and exported using a built-in program. Thus, with this tool, investigators can assess specific GCIPL parameters in a wide area of the macula and evaluate GCIPL changes in a particular region of the macula.

The purpose of the present study was to evaluate the glaucoma diagnostic ability of the GCIPL thickness in various areas centered on the fovea using wide-angle SS-OCT. In addition, we validated the various GCIPL measurements by comparing their diagnostic utility with that of a conventional GCIPL measurement method and correlating the outcomes to VF defects in patients with glaucoma.

## Methods

This was a prospective, cross-sectional, comparative study. The study was conducted in compliance with the Health Insurance Portability and Accountability Act and adhered to the tenets of the Declaration of Helsinki, and ethics approval was obtained from the Institutional Review Board (IRB) of Pusan Nation University Yangsan Hospital (IRB #05–2019-005).

Study participants were patients with glaucoma who underwent medical glaucoma treatment and age-matched normal controls who visited our clinic for regular health examinations for refractive errors. Glaucoma was defined with the following criteria: asymmetric cup-to-disc ratio ≥ 0.2, vertical cup-to-disc ratio > 0.7, neural rim thinning, localized notching, disc hemorrhage and retinal nerve fiber layer (RNFL) defects with corresponding glaucomatous VF defects. Patients with open-angle glaucoma detected by gonioscopic examination and normal controls with no history of ocular diseases (intraocular pressure (IOP) ≤ 21 mmHg), an absence of a glaucomatous optic disc, and a normal VF were recruited. Subjects were excluded if they had a best-corrected visual acuity (BCVA) of less than 20/40; a refractive error beyond the range from − 6.0 to + 3.0 diopters; an astigmatism of more than ±3.0 diopters; and a history of ocular trauma, ocular surgery, laser treatment, or ocular and/or systemic disease that could affect the optic nerve or VF.

All participants underwent a complete ophthalmologic examination, which included BCVA measurement, slit-lamp examination, axial length assessment, gonioscopy, dilated fundus examination, and stereoscopic optic disc photography. An automated VF examination was also performed on all subjects with a standard 24–2 Swedish interactive thresholding algorithm (SITA) program on a Humphrey 740 Visual Field Analyzer (Carl Zeiss Meditec, Dublin, CA, USA). We defined glaucomatous VFs on the basis of the presence of two of the following criteria: (1) an abnormal glaucoma hemifield test result (a borderline score was not considered abnormal); (2) three continuous non-edge points (allowing for two-step nasal edge points) with *P* <  0.05 on the total deviation plot, with at least one point with a *P*-value < 0.01; and (3) P <  0.05 for the pattern standard deviation (PSD) on the SITA standard test.

Wide-angle scanning using an SS-OCT device (DRI-OCT-1 Atlantis; Topcon, Tokyo, Japan) was performed on each subject. Wide-angle scanning uses a wide-angle 12 × 9 mm lens, with the scan centered on the fovea, for 256 B-scans, each comprising 512 A-scans, for a total of 131,072 axial scans per volume. A scan time of 1.3 s per 12 × 9 mm^2^ scan, which was previously shown to be sufficient for acquiring all images, [7] was used here. Poor-quality images (image-quality scores less than 50, poorly focused, or decentered during fovea scanning) or those acquired after segmentation failures or with artifacts due to eye movements or blinking were excluded. The built-in DRI-OCT-1 software (version 9.12) automatically identified the outer boundary of the RNFL, from the internal limiting area to the retinal ganglion cells and the outer boundary of the IPL. The difference between the RNFL and the inner plexiform layer (IPL) outer boundary yielded the combined GCIPL thickness.

**Fig. 1:**
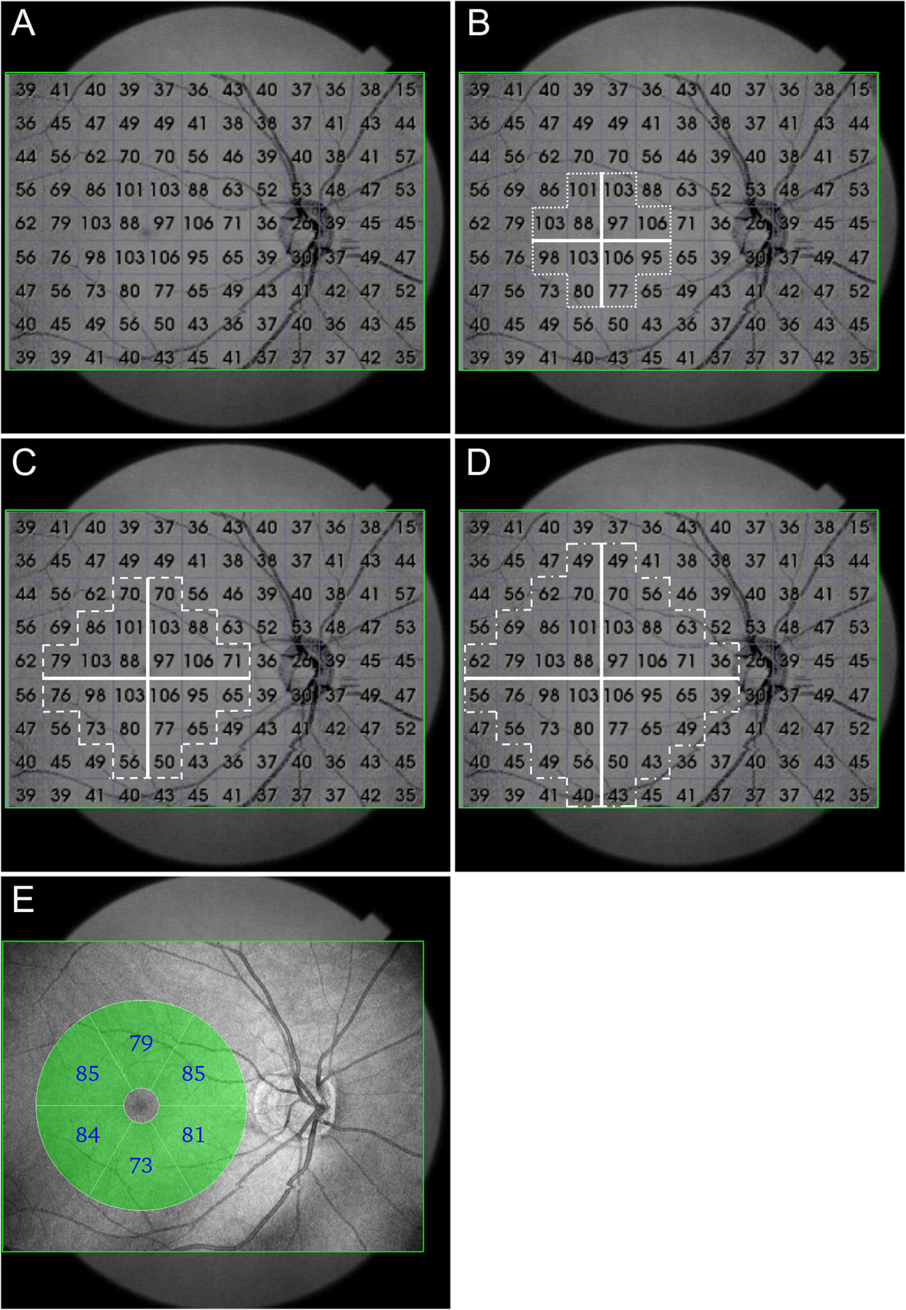
**a** A wide-angle scan with swept-sources optic coherence tomography (SS-OCT; DRI-OCT-1 Atlantis; Topcon, Tokyo, Japan) demonstrating the ganglion cell-inner plexiform layer (GCIPL) thickness for each 1-mm^2^ grid square of a 12 mm × 9 mm (horizontal × vertical) scan; and these 108 data were collected (green rectangles). We made zone classifications based on the scanned area (centered on the fovea) and the arcuate configuration of the papillomacular bundle. **b** Zone 1 (narrow area); the widest horizontal × vertical scanned length was 4 × 4 mm, with a total of 12 grid squares and 3 squares per quadrant. **c** Zone 2 (mid-sized area); the widest horizontal × vertical scanned length was 6 × 6 mm, with a total of 24 squares with 6 per quadrant included. **d** Zone 3 (wide area); the widest horizontal X vertical scanned length was 8 × 8 mm, and a total of 40 squares with 10 squares per quadrant were included. **e** Conventional area; 6 × 6 mm annulus area centered on the fovea

For each wide-angle scan, DRI-OCT-1 software was used to calculate the GCIPL of each 1-mm^2^ grid square across the 12 × 9 mm^2^ scan, yielding 108 data points that were displayed and exported using a built-in program (Fig. 1a). We developed a zonal classification system based on the scanned area centered on the fovea that reflected the arcuate configuration of the papillomacular bundle. The zones were defined as follows: zone 1 (narrow area) contained a maximum horizontal and vertical scanned width and length, respectively, of 4 mm and a total of 12 grid squares with 3 squares per quadrant (Fig. 1b); zone 2 (mid-sized area) contained a maximum horizontal and vertical scanned width and length, respectively, of 6 mm and a total of 24 grid squares with 6 squares per quadrant (Fig. 1c); zone 3 (wide area) contained a horizontal and vertical scanned width and length, respectively, of 8 mm, and a total of 40 grid squares with 10 squares per quadrant (Fig. 1d); and the conventional area contained a 6 × 6 mm^2^ annulus centered on the fovea but excluding the inner 1 × 1 mm^2^. In addition, we assessed the following GCIPL parameters for each zone. 1) the quadrant GCIPL thickness: the average GCIPL thickness in the superotemporal, superonasal, inferotemporal, and inferonasal areas; 2) the average thickness: the average of the total grids in the zone; and 3) the minimum GCIPL thickness: the grid with the thinnest GCIPL thickness in the zone. We obtained GCIPL parameters within a 6 × 6 mm^2^ annulus area centered on the fovea using conventional, automated Cirrus HD-OCT software GCA algorithms.

We obtained three images on the same visit day; two of the three GCIPL parameters were assessed using wide-angle SS-OCT twice to evaluate intraobserver agreement. The half- width of the 95% limits of agreement and intraclass correlation coefficients (ICCs) were calculated. We investigated correlations and agreement among the 6-mm-diameter GCIPL parameters derived via the SD-OCT technique, which were based on the Early Treatment Diabetic Retinopathy Study (ETDRS) area and GCIPL parameters from zone 2. Bland-Altman plots were constructed, and Pearson’s correlation coefficients were calculated to analyze correlations and agreements. We evaluated the diagnostic utility of the GCIPL measurements from zone 1, zone 2, and zone 3 for differentiating glaucoma from normal eyes. We also used GCIPL parameters in each quadrant from zones 1, 2, and 3 and constructed receiver operating characteristic (ROC) curves to analyze the diagnostic accuracies of the newly developed method. The area under the ROC (AUROC) curve was calculated to assess the diagnostic accuracy of each measurement. An AUROC of 1.0 represented perfect discrimination, while an AUROC of 0.5 represented discrimination due to chance. The method described by DeLong et al. was used to compare the AUROCs.

Data normality was assessed with the Kolmogorov-Smirnov test. Student’s t-tests or Mann-Whitney U tests were used to compare continuous data. *P*-values < .05 were considered to indicate statistical significance. Statistical analyses were performed using SPSS for Windows 21.0 (SPSS, Inc., Chicago, IL) and Medcalc version 10.0 (Medcalc Software; Ostend, Mariakerke, Belgium).

## Results

This study included 61 glaucomatous eyes from 61 patients and 59 normal eyes from 59 healthy individuals. The participants’ demographic data are summarized in Table 1. There were no significant differences in sex, age, spherical equivalent, axial length, or central corneal thickness (CCT) between the two groups, although there were significant differences in mean deviation (MD) (*P* <  0.001).

**Table 1:** Demographic characteristics in the present study

Parameters	Normal control	Glaucoma group	*P*-value
Age	54.22 ± 11.91	54.82 ± 10.06	0.766 ^a^
Sex (Male/Female)	33/26	30/31	0.471 ^b^
Central corneal thickness, μm	533.18 ± 41.15	525.12 ± 35.05	0.514 ^a^
Spherical equivalent	- 0.92 ± 2.37	- 0.49 ± 2.18	0.313 ^a^
Axial length	23.93 ± 1.06	23.97 ± 1.15	0.856 ^a^
Average RNFL thickness, μm	103.90 ± 1.81	66.58 ± 3.21	< 0.001 ^a^
Humphrey 24–2 visual field			
MD, dB	- 0.42 ± 0.95	- 8.14 ± 5.01	< 0.001 ^c^
PSD, dB	1.56 ± 0.45	8.61 ± 4.11	< 0.001 ^c^
VFI, %	99.18 ± 1.09	78.98 ± 16.15	< 0.001 ^c^

*RNFL* Retinal nerve fiber layer, *MD* mean deviation, *PSD* pattern standard deviation, *VFI* visual field index

^a^ Student t-test, ^b^ Fisher’s exact test, ^c^ Mann-Whitney U test

The sectorial, minimum, and average GCIPL measurements in zones 1, 2, and 3 as well as the conventional 6 × 6 mm GCIPL thicknesses significantly differed among the normal and glaucoma cohorts (*P* <  0.001) (Table 2). The GCIPL thickness parameters in the glaucomatous eyes were smaller than those in the eyes of normal controls.

**Table 2:** Comparison of GCIPL thickness between normal an glaucoma using SS-OCT

Parameters (μm)	Normal	Glaucoma	*P*-value ^a^
In conventional 6 × 6 mm			
Superonasal sector	72.76 ± 7.33	64.90 ± 10.09	< 0.001
Superior sector	67.79 ± 6.89	59.00 ± 9.31	< 0.001
Superotemporal sector	72.42 ± 4.91	62.67 ± 8.13	< 0.001
Inferonasal sector	69.88 ± 7.47	60.78 ± 9.53	< 0.001
Inferior sector	64.45 ± 5.84	54.32 ± 7.13	< 0.001
Inferotemporal sector	71.37 ± 5.20	58.98 ± 6.53	< 0.001
Average	69.78 ± 5.14	60.11 ± 7.07	< 0.001
Minimum	63.69 ± 6.10	51.90 ± 7.09	< 0.001
In Zone 1			
Superonasal sector	84.18 ± 5.86	73.34 ± 13.49	< 0.001
Superotemporal sector	78.51 ± 5.25	68.03 ± 11.47	< 0.001
Inferonasal sector	79.20 ± 6.36	64.81 ± 11.04	< 0.001
Inferotemporal sector	80.36 ± 5.78	61.90 ± 9.83	< 0.001
Average	80.56 ± 5.23	67.02 ± 9.87	< 0.001
Minimum	76.61 ± 5.07	59.15 ± 9.70	< 0.001
In Zone 2			
Superonasal sector	68.83 ± 5.02	60.89 ± 9.29	< 0.001
Superotemporal sector	70.56 ± 4.81	61.90 ± 8.01	< 0.001
Inferonasal sector	60.42 ± 5.26	52.12 ± 6.35	< 0.001
Inferotemporal sector	65.18 ± 5.21	55.31 ± 4.86	< 0.001
Average	66.25 ± 4.35	57.56 ± 5.96	< 0.001
Minimum	60.22 ± 5.11	51.23 ± 6.25	< 0.001
In Zone 3			
Superonasal sector	59.58 ± 4.67	52.86 ± 7.04	< 0.001
Superoteomporal sector	62.98 ± 4.23	56.55 ± 6.28	< 0.001
Inferonasal sector	52.11 ± 4.56	46.29 ± 5.22	< 0.001
Inferotemporal sector	56.78 ± 4.51	51.48 ± 4.32	< 0.001
Average	57.86 ± 3.82	51.79 ± 4.66	< 0.001
Minimum	51.95 ± 4.40	45.66 ± 5.17	< 0.001

^a^ Student t-test

Since zone 2 is similar to the ETDRS area, which was defined as the 6-mm-diameter area centered on the fovea, we analyzed correlations and agreements between the GCIPL parameters from zone 2 and the ETDRS area. The ICCs of the OCT measurements in the superior and inferior areas and the minimal and average measurements in zone 2 were 0.874, 0.882, 0.858, and 0.862, respectively, in the glaucoma group and 0.899, 0.915, 0.928, and 0.958, respectively, in the normal group. The GCIPL measurements in the superior and inferior areas and the minimal and average measurements showed a significantly positive correlation between the conventional 6 × 6 mm area and zone 2 based on Pearson’s correlation coefficient in both the normal controls (r = 0.805, 0.746, 0.834, and 0.580, respectively) and the glaucoma patients (r = 0.886, 0.879, 0.953, and 0.791, respectively), as illustrated in Table 3.

**Table 3:** Univariate correlation of GCIPL thickness in between automated 6 × 6 mm and zone 2

Parameters	Normal	Glaucoma	Total
Superior sector	0.805^*^	0.886^*^	0.901^*^
Inferior sector	0.746^*^	0.879^*^	0.899^*^
Average	0.834^*^	0.953^*^	0.948^*^
Minimum	0.580^*^	0.791^*^	0.836^*^

* *P* < 0.001 by Pearson correlation analysis

A Bland-Altman analysis that compared each GCIPL measurement between the conventional 6 × 6 mm^2^ area and zone 2 revealed differences in the mean thicknesses of the two areas (Table 4). There were no significant correlations between the parameter differences and the mean GCIPL thicknesses for all subjects. In both the normal and glaucoma groups, there was reasonable agreement of the superior, inferior, average and minimum GCIPL thickness between zone 2 and the conventional 6 × 6 mm^2^ area.

**Table 4:** Bland-Altman Analysis of GCIPL measurements with each parameter between automated 6 × 6 mm and zone 2

	95% LoA in Normal			95% LoA in Glaucoma		
	Lower	Upper	Width	Lower	Upper	Width
Superior sector	- 7.8	5.2	13.0	−8.9	7.4	16.3
Inferior sector	- 12.7	1.2	13.9	- 11.0	2.4	13.4
Average	- 9.1	2.0	11.1	- 6.9	1.8	8.7
Minimum	- 14.0	7.1	21.1	- 8.2	6.9	15.1

Table 5 shows the AUROCs for each sectorial, minimum, and average GCIPL parameter from the conventional area and zones 1, 2, and 3. Among the GCIPL parameters in the conventional area, the GCIPL thickness in the inferotemporal sector and the minimum GCIPL thickness showed the best diagnostic ability (AUROCs: 0.920 and 0.908, respectively), but these parameters in the superonasal sector showed a poor diagnostic ability (AUROC: 0.737). Among the parameters in zone 1, the GCIPL thickness in the inferotemporal sector and the minimum GCIPL thickness also showed the best diagnostic ability (AUROCs: 0.931 and 0.945, respectively), but in the superonasal sector, these parameters showed a poor diagnostic ability (AUROC: 0.764). Among the parameters in zone 2, the GCIPL thickness in inferotemporal sector showed the best diagnostic ability (AUROC: 0.918), but these parameters in the superionasal sector also showed poor diagnostic ability (AUROC: 0.770). Among the parameters in zone 3, the average GCIPL thickness showed the best diagnostic ability (AUROC: 0.840), but the superonasal sectorial GCIPL thickness showed a poor diagnostic ability (AUROC: 0.777). In addition, the minimum GCIPL in zone 1 most accurately allowed discrimination between patients with and without glaucoma.

**Table 5:** Area under the receiver operating characteristic curves of GCIPL parameters between normal and glaucoma

Parameters	Mean (SD)
In conventional 6 × 6 mm	
Superonasal sector	0.737 (0.047)
Superior sector	0.788 (0.043)
Superotemporal sector	0.850 (0.035)
Inferonasal sector	0.801 (0.041)
Inferior sector	0.870 (0.027)
Inferotemporal sector	0.920 (0.027)
Average	0.872 (0.033)
Minimum	0.908 (0.029)
In Zone 1	
Superonasal sector	0.764 (0.044)
Superotemporal sector	0.778 (0.043)
Inferonasal sector	0.871 (0.032)
Inferotemporal sector	0.931 (0.024)
Average	0.894 (0.028)
Minimum	0.945 (0.019)
In Zone 2	
Superonasal sector	0.770 (0.044)
Superotemporal sector	0.822 (0.039)
Inferonasal sector	0.839 (0.036)
Inferotemporal sector	0.918 (0.024)
Average	0.879 (0.031)
Minimum	0.865 (0.032)
In Zone 3	
Superonasal sector	0.777 (0.042)
Superotemporal sector	0.817 (0.039)
Inferonasal sector	0.800 (0.041)
Inferotemporal sector	0.809 (0.040)
Average	0.840 (0.036)
Minimum	0.821 (0.039)

The parameter of minimum and inferotemporal sector in conventional 6 × 6 mm and zone 1, and inferotemporal sector measurement in zone 2 which have occupied an area of 0.9 or more have shown higher diagnostic value than other GCIPL parameters

**Fig. 2:**
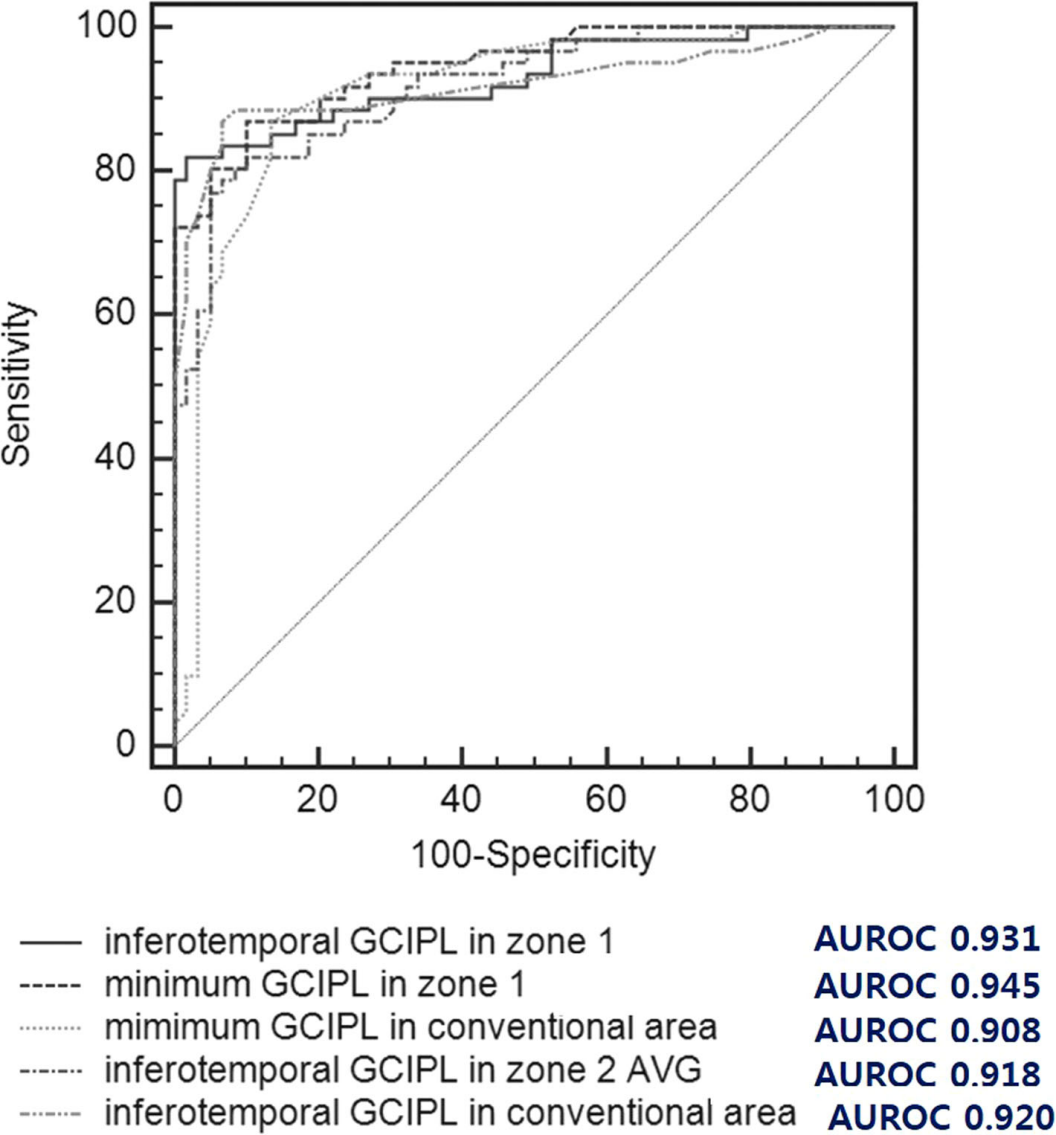
Receiver operating characteristic (ROC) curves for the minimum ganglion cell-inner plexiform layer (GCIPL) measurements of zone 1 and conventional area and the GCIPL thickness of the inferotemporal area in zone 1, zone 2, and the conventional area. These curves show that the areas under the receiver operating characteristic (AUROCs) curves were greater than 0.9

Figure 2 shows the ROC curves of the inferotemporal and minimum GCIPL thicknesses in the conventional area, the inferotemporal and minimum GCIPL thicknesses in zone 1, and the inferotemporal GCIPL thickness in zone 2, whose areas were all greater than 0.9 (AUROCs: 0.920, 0.908, 0.931, 0.945, and 0.918, respectively). The diagnostic values of the inferotemporal GCIPL and the minimum GCIPL parameters in zone 1 were compared to those of the inferotemporal, average and minimum GCIPL thicknesses in the conventional area and zone 2 in each eye with glaucoma. Notably, the AUROCs for both the inferotemporal and minimum GCIPL thicknesses in zone 1 were significantly greater than those of the average GCIPL thickness in zone 2 (*P* = 0.041 and *P* = 0.005), the average thickness of the conventional area (*P* = 0.045, *P* = 0.004), and the minimum thickness of the conventional area (*P* = 0.018 and *P* <  0.001) in eyes with glaucoma (Table 6).

**Table 6:** Pairwise comparison of area under the receiver operating characteristic of GCIPL thickness in glaucoma group conducted by DeLong et al.’s method

Difference between areas	Inferotemporal sector of zone 1	Minimum of Zone 1
Inferotemporal sector of zone 2	0.011 *(0.394)*	0.024 *(0.261)*
Average of zone 2	0.058^*^ *(0.041)*	0.072^*^ *(0.005)*
Minimum of zone 2	0.023 *(0.443)*	0.036 *(0.163)*
Inferotemporal sector of conventional 6 × 6 mm	0.013 *(0.434)*	0.027 *(0.111)*
Average of conventional 6 × 6 mm	0.051^*^ *(0.045)*	0.066^*^ *(0.004)*
Minimum of conventional 6 × 6 mm	0.066^*^ *(0.018)*	0.079^*^ *(< 0.001)*

() *P* value by methods described in DeLong et al, ^*^ *P* value < 0.05

## Discussion

The GCIPL thickness parameters in the present study were not only significantly correlated with conventional ETDRS parameters, but also significantly differed between patients with and without glaucoma. Importantly, the minimum GCIPL in zone 1 around the fovea showed the best diagnostic value for glaucoma among the various GCIPL parameters that were investigated in the present study.

Previous studies have demonstrated the topographic pattern of the retinal ganglion cell density [8–12]. In normal eyes, the thickness of the ganglion cell layer originating in the ganglion cell density sharply increases from the central fovea, with a peak height approximately 1 mm from the fovea; it then steadily decreases as the distance from the fovea increases. Kerrigan-Baumarind et al. obtained retinal ganglion cell density estimates at the same test points used in the Humphrey 30–2 test. This study found that the retinal ganglion cell density in a normal retina was nearly 10 times higher 6 degrees from the central fixation point than at more peripheral points [13]. Another retinal ganglion cell study that used multifocal visual evoked potentials found that reductions in the amplitude width appeared to be greater in the parafoveal region than at more peripheral locations in eyes with glaucoma [14, 15].

Given the results discussed above, we speculate that the diagnostic ability of the GCIPL parameter would increase as its measurements become closer to the fovea. Prior studies have found that the GCIPL thickness 7.2 degrees outside of the retina was not sufficient to diagnose glaucoma, as the retinal ganglion cell layer thickness decreases as one moves away from the macula [16–18]. The GCIPL located around the fovea might be more susceptible to damage from glaucoma, as shown by the GCIPL thickness findings reported here. Similarly, Raza et al. reported that the relationship between the VF defect region and GCIPL thickness is stronger within 7.2 degrees of the retina, which corresponds to approximately 6 degrees of the central VF [18]. In addition, quantitative analysis performed in a previous study showed that the correlation between the GCIPL thickness within 7.2 degrees of the retina and the corresponding regions of VF defects was higher than the correlation outside this region. Furthermore, since the GCIPL parameters in zone 1 (based on a 4 × 4 mm^2^ area around fovea) might have a stronger relationship with VF defects than those within 6 degrees of the fovea, further study will be needed to evaluate the structure-function relationship.

The present study found that the GCIPL parameters in zone 1 could be reliably used for the diagnosis of glaucoma. In agreement with previous studies, the present study also revealed that the minimum GCIPL thickness was the most accurate parameter for diagnosing glaucoma [19–21]. Similarly, Jeong et al. proposed that glaucoma progression occurs focally, and the susceptibility of retinal ganglion cells to glaucomatous damage differs across regions [19]. Thus, the average or sectorial GCIPL thickness might not be able to detect glaucomatous damage as sensitively as the minimal GCIPL thickness.

The inferotemporal GCIPL parameters in the present study also showed high AUROCs for the detection of glaucoma. These results are in agreement with prior results, which suggested that the inferotemporal area of the macula is the most vulnerable papillomacular bundle area to glaucomatous damage [14, 17, 19, 22, 23]. Furthermore, Hood et al. speculated that the vulnerable area to damage in glaucoma is a part of the high-density axon region and that the probability of glaucomatous damage is proportional to the axon density, which is known as the crowding hypothesis [3, 17, 18]. Takayama et al. similarly proposed that the papillomacular bundle is typically spared until advanced glaucoma damage occurs, and that the retinal ganglion cells in the nasal macular area are less susceptible to glaucoma than those in the temporal side to the macula [14].

With decreasing GCIPL thickness, the GCIPL parameters in OCT become more sensitive to glaucoma-related changes and thus more diagnostically useful. The minimum GCIPL thickness in zone 1, based on a 4 × 4 mm^2^ area centered on the fovea, was the most accurate parameter for the detection of glaucoma among all of the GCIPL parameters assessed here across wider areas that were also centered on the fovea. Since nearly complete retinal images could be obtained through wide-scanning techniques in SS-OCT or SD-OCT, this technique is needed to investigate various parameters based on SS-OCT images.

There are some limitations in the present study. First, the sample size of the study was relatively modest. Second, in the wide-scan images, the fovea was not located in the same place in each participant, and the variability in identifying the grid around the fovea could lead to errors in the results. To minimize the variation of the fovea in the scan, we compared the parameters in the superior and inferior areas between each zone 2 and conventional ETDRS area. The data in the present study showed the reliable correlation and agreements between two areas. Third, the SS-OCT 12 × 9 mm^2^-wide scan protocol used here enabled us to analyze the GCIPL thickness around the macula in only a square 1 × 1 mm^2^ region, rather than in a circular 1 × 1 mm^2^ region. As such circular measurements are not possible with the present SS-OCT protocol, the present study based on square area analysis was limited in its ability to evaluate the diagnostic value of the GCIPL thicknesses in a circular area around the macula. However, to minimize the bias associated with using a square area for measuring the GCIPL thickness, we compared measurements from the conventional 6 × 6 mm^2^ area with those obtained using our newly developed approach, which analyzes a square 6 × 6 mm^2^ area. We found that the GCIPL thickness of the newly created area did not differ significantly from that found in the conventional 6 × 6 mm^2^ area. Fourth, the participants in the present study underwent only standard 24–2 VF examination, not 10–2 VF test as functional test. Since the 10–2 visual field test has been useful tool to detect central visual dysfunction, the further investigation will be needed to evaluate the relationship between the various GCIPL parameters according to the range around the fovea and the functional damage detected by the 10–2 VF test.

## Conclusions

The present study suggests that the minimum GCIPL thickness in a narrower area has the better diagnostic value for the discrimination between glaucoma and normal eyes than the GCIPL parameters acquired from wider areas centered on the fovea. We expect that improvements in SS-OCT image acquisition protocols will contribute to improvements in the detection of glaucomatous damage using SS-OCT.

## Data Availability

The datasets used and/or analysed during the current study are available from the corresponding author on reasonable request.

## Abbreviations

AUROCs: Areas under the receiver operating characteristic
BCVA: Best-corrected visual acuity
CCT: Central corneal thickness
ETDRS: Early Treatment Diabetic Retinopathy Study
GCIPL: Ganglion cell-inner plexiform layer
ICCs: Intraclass correlation coefficients
IOP: Intraocular pressure
IPL: Inner plexiform layer
IRB: Institutional Review Board
MD: Mean deviation
PSD: Pattern standard deviation
RNFL: Retinal nerve fiber layer
ROC: Receiver operating characteristic
SD-OCT: Spectral domain optical cohrerence tomography
SITA: Swedish interactive thresholding algorithm
SS-OCT: Swept-source optical cohrerence tomography
VF: Visual field
